# A robust iterative algorithm for image restoration

**DOI:** 10.1186/s13640-017-0201-6

**Published:** 2017-08-03

**Authors:** Yuewei Liu, Weiping Lu

**Affiliations:** 1 School of Mathematics and Statistics, Lanzhou University, Lanzhou, China; 2 School of Engineering and Physical Sciences, Heriot Watt University, Edinburgh, UK

**Keywords:** Image restoration, Ill-posed problem, Iterative cost function, Regularized gradient, Noise reduction filter, Residual optimization

## Abstract

We present a new image restoration method by combining iterative VanCittert algorithm with noise reduction modeling. Our approach enables decoupling between deblurring and denoising during the restoration process, so allows any well-established noise reduction operator to be implemented in our model, independent of the VanCittert deblurring operation. Such an approach has led to an analytic expression for error estimation of the restored images in our method as well as simple parameter setting for real applications, both of which are hard to attain in many regularization-based methods. Numerical experiments show that our method can achieve good balance between structure recovery and noise reduction, and perform close to the level of the state of the art method and favorably compared to many other methods.

## Introduction

Image restoration aims to compensate for or undo the defects that degrade an image. Degradation can come in many forms such as motion blur, noise, and camera defocus. In optical microscopes, there are predominately two sources for degradation in the imaging systems, blurring and noise, which can be described by the general imaging model

1 $$ J={{PI}}+N,  $$

where *I,J* are the ground truth and the corresponding observation, respectively, *P* is a point spread function (PSF), and *N* is a noise which is assumed to be independent to the ground truth. The simplest way to estimate the ground truth from the observation is by minimizing the residual,

2 $$ \text{min}{\left \| J-{{PI}} \right \|}_{2}^{2},  $$

which can lead to the least square solution. Unfortunately, a unbounded noise will be introduced into the solution because the PSF matrix always has small eigenvalues even it is invertible. This is not surprising as (2) is well known to be ill-posed [1].

There are now vast literatures to tackle the problem of image restoration. A recent trend is concentrated on a sparse block matching 3-D (BM3D)-based restoration technique. BM3D algorithms are initially developed for collaborative filtering through a non-local modeling of images by collecting similar image patches in 3D arrays [2]. They have recently been incorporated into image restoration for solving regularized inverse problems for image denoising as well as deblurring [3]. Another development based on BM3D is sparse representation for image restoration, where the image is considered to be a combination of a few atomic functions taken from a certain dictionary and can be parameterized and approximated locally or non-locally by these functions [4]. The dictionary is usually considered as an over-complete system in order to better describe all variety of images. There are now many published works on the sparsity-based models and methods [5]. For example, the formulation of IDD-BM3D image modeling in terms of the over-complete sparse frame representation for image reconstruction has led to impressive restoration performance [6]. This approach allows decoupling between deblurring and denoising by considering the optimization problem as a generalized Nash equilibrium balance of two objective functions. A distinct advantage of this approach is that various denoising algorithms can be selected independently with respect to deblurring algorithms, which have demonstrated better performance than those where deblurring and denoising are jointly performed in many cases. However, for the decoupled algorithms such as [6], [7], and [8], the parameters setting for optimal performance of regularization is usually complicated and reasons for the best setting are often not explained. Another shortage of these methods is the lack of error analysis for the solutions because of the complexity of the regularization factors.

In this paper, we present a new image restoration method based on the inverse operator theory. As we know, the inverse operator theory [9] gives the solution of *P* ^−1^ *J*=*I*+*P* ^−1^ *N* for the general imaging model (1), where *P* ^−1^ is the inverse or pseudo-inverse of the PSF matrix. Due to small eigenvalues of *P, P* ^−1^ *N* leads to significant noise amplification to the ground truth. To overcome this problem, we propose a new approach that combines iterative VanCittert algorithm with noise reduction modeling, the latter enables to reliably estimate the gradient in the presence of noise so that the VanCittert iteration can converge to the ground truth even when the observation is noise contaminated. This work has several contributions to the research area of image restoration. Firstly, it extends the inverse operator theory to image restoration in the presence of noise, which offers a different approach to that of the present popular regularization methods. Secondly, our method enables decoupling between deblurring and denoising, so any well-established noise reduction operator can be selected in our model, independent of the VanCittert deblurring operation. Thirdly, our approach allows error analysis of the solutions because the structure recovery and noise amplification in the VanCittert iterations can be separated analytically, which is an advantage over many regularization methods for which errors are difficult to be estimated due to complicated regularization factors. Finally, parameter setting in our method is simple and robust to the performance. There are only two parameters in our method: *σ*, as the noise reduction strength, and *s*, the interval between two neighboring denoising operations. We have further developed an automated parameter setting procedure for our method, which has no need to set the parameters manually. The above points have been verified by numerical experiments, which also show that our method performs close to the level of the state of the art method and favorably compared to many other methods.

## Methods

Our method is motivated by the iterative VanCittert algorithm, which has a long history as a simple and efficient approach for image restoration. The algorithm is formulated for spatially invariant or variant restoration problems with neglect of noise contribution in (1). Originally, it is a steepest descent method but the solution does not converge if the step parameter is assumed to be real values. To overcome this shortage, an iterative procedure was proposed [10],

3 $$ I_{k}=I_{k-1}+\beta P^{T}(J-{PI}_{k-1}),  $$

which converges to the ground truth only if noise in an observation is negligible, where *P* ^*T*^ is the transpose of *P*. When an observation comprises noise, VanCittert iteration (3) can be expressed as [9, 11, 12]

4 $$\begin{array}{*{20}l} I_{k}&=\sum\limits_{u,v}\left(1-\left(1-\beta|\zeta_{{uv}}|^{2}\right)^{k}\right)\left(I,Z_{{uv}}\right)Z_{{uv}}  \\ &\quad+\sum\limits_{u,v}\frac{1}{\zeta_{{uv}}}\left(1-\left(1-\beta|\zeta_{{uv}}|^{2}\right)^{k}\right)\left(N,Z_{{uv}}\right)Z_{{uv}}\\ &\quad\text{for}\ k=1,2,\ldots, \end{array} $$

where {*ζ*_*uv*_:*u*=1,2…,*R,v*=1,2,…,*C*} and *Z*_*uv*_ are the eigenvalues and eigenvectors of *P*, and *R*,*C* are image size, and *u*,*v* the indices of image pixels and *β* the step parameter. The first term involving *I* describes structure recovery while the second term involving *N* shows noise amplification, so structures and noise are separated in (4). For a noisy observation, however, small eigenvalues *ζ*_*uv*_ can lead to significant noise amplification in the second term of (4) so the inverse problem becomes ill-posed. Therefore, we have to suppress the noise in the second term if the iteration gives any hope to converge to the ground truth.

To tackle the above problem, we first introduce a noise reduction operator, **Ψ**, which minimizes the estimation error of a cost function. Letting *I* be the ground truth, *N* be a white noise and *V*=*I*+*N*, we define the cost function for the noise reduction operator,

$$ \mathcal{C}(\mathbf{\Psi},I)=\mathrm{E}\left\{{\left \| I-\mathbf{\Psi}(V) \right \|}_{2}^{2}\right\}, $$

where E{·} is the expectation taken over the noise distribution. The error is measured by *L*_2_ norm and averaged over the noise distribution. For the general imaging model (1), we propose our method as


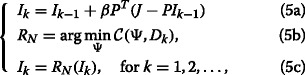


where $D_{k}={\sum \nolimits }_{u,v}\left (1-\left (1-\beta |\zeta _{{uv}}|^{2}\right)^{k}\right)(I,Z_{{uv}})Z_{{uv}},$ is the first term in (4). As seen from (5), the VanCittert iteration (5a) tends to recover the structures by searching a solution along the gradient of (2). However, the gradient is contaminated by noise, leading to noise amplification in the iterative solution. A noise reduction operation (5b) and (5c) is then applied to remove noise and to optimize the gradient for the next iteration.

As for noise reduction of (5b), our method do not expect an ideal operator removing all noise [13]. Instead, it can be any denoising algorithm as long as the operator satisfies the following condition,

6 $$ R_{N}(I+N)=I+o(N) \ \text{and}\ \text{Var}(o(N))\propto\Delta \sigma^{2},  $$

where *σ* ^2^=Var(*N*) is the variances of noise *N* and *Δ*≪1 is the noise reduction factor. The condition (6) implies that remaining noise *o*(*N*) has a variance far less than the initial noise *N* after applying the operator *R*_*N*_ on a noisy image. We will show in error analysis below that when the condition (6) is satisfied, the iterative solution of (5) converges to the ground truth with a higher order small noise term, i.e., *I*_*k*→*∞*_=*I*+*o*(*N*). A necessary condition for the iterative process (5) to converge is that |1−*β*|*ζ*_*uv*_|^2^| must fall within [0,1) for all the eigenvalues of *P*, which leads to

7 $$ 0<\beta \leq \text{min}_{u,v}\frac{2}{|\zeta_{{uv}}|^{2}}.  $$

Since most of PSFs act as a low-pass filter, the maximum absolute value of their eigenvalues is about 1. This means that it is easy to set a value for *β* that satisfies condition (7) and the following condition:

8 $$ {0\leq (1-\beta|\zeta_{{uv}}|^{2})<1 \text{for all the eigenvalues.} }  $$

For example, we set *β*=1 in our experiments.

To implement the method (5), we can apply any well-established noise reduction algorithms to combine with the VanCittert iteration, for example, the wavelet domain shrinking filter $T_{{SW}}(V,\delta)=\hat {I}=Ww,$ where $\hat {I}$ is the estimated image, *W* is a group of wavelet bases and *w* is a vector of shrinking coefficients depending on the smooth parameter *δ* [13]. The smooth parameter *δ* can be determined in a similar form to (5b) by

$$ \arg\min_{\delta}\mathcal{C}(T_{\text{SW}}(V,\delta),I), $$

which has a noise shrinkage strength of *Δ*=(2logRC+1)(logRC+1)/RC, where *R* and *C* are the image size. For images of modest size, *Δ*≪1 so the wavelet algorithm satisfies (6). Another popular denoising method is the state of the art BM3D method. BM3D improves from wavelet domain shrinking by incorporating the concept of image patches and non-local mean (NLM) [14] into a transformed domain and has shown the highest peak signal-to-noise ratio in its performance compared to the wavelet domain and other algorithms. Moreover, BM3D has simple parameter setting and is easy to use. Mathematically, BM3D can be expressed as

9 $$ O_{\mathrm{BM3D}}= \mathcal{A}{\mathcal{T}_{\mathrm{3D}}}^{-1}W_{\text{wie}}\mathcal{T}_{\mathrm{3D}}Z,  $$

where *Z* is the stacked noisy blocks, $\mathcal {T}_{\mathrm {3D}}$ is the transformation from spatial domain to frequency domain with discrete cosine bases, and *W*_wie_ is the Wiener shrinkage operator and $\mathcal {A}$ is the aggregation operator, all defined in [2]. In view of the advantage and excellent performance of BM3D [2], we choose the operator *R*_*N*_=*O*_BM3D_ in our method (5) for the numerical experiments below.

We note that while we follow the same decoupled approach for deblurring and denoising as IDD-BM3D [6], our method has two advantages. Firstly, structure restoration by the VanCittert algorithm in (5) has a simple step parameter of *β*=1, while the regularization factors for optimal deblurring in [6] and [7] are much more complex to set. This leads to overall simple parameter setting of our method compared to many regularization methods. Secondly, structure and noise can be separated analytically in (5), which allows us to perform error analysis for a restored image, while error analysis for regularization methods is generally hard to attain due to the complexity of the regularization factors.

### Error analysis

Given (4), the noise amplification is separated from structures so VanCittert algorithm allows error analysis for our method. We begin with the following lemma and then give theorem (1).

#### **Lemma 1**

Let *F*_*k*_=1/*ζ*_*uv*_(1−(1−*β*|*ζ*_*uv*_|^2^)^*k*^) be the noise amplification factor in Eq. (4), then 1≤*F*_*k*_/*F*_*k*−1_<2 for *β* satisfying (8) and *k*≥2.

#### *Proof*

For convenience we set *a*=(1−*β*|*ζ*_*uv*_|^2^), thus *F*_*k*_=1/*ζ*_*uv*_(1−*a* ^*k*^). Because of condition (8) *a* drops in [0,1). The ratio

$$\begin{array}{*{20}l} \frac{F_{k}}{F_{k-1}} & =\frac{1-a^{k}}{1-a^{k-1}}=\frac{1+a+a^{2}+\cdots+a^{k-1}}{1+a+a^{2}+\cdots+a^{k-2}} \\ & =1+\frac{a^{k-1}}{1+a+a^{2}+\cdots+a^{k-2}}, \end{array} $$

so 1≤*F*_*k*_/*F*_*k*−1_<2 for any *a*∈[0,1) when *k*≥2. □

#### **Theorem 1**

For any operator *R*_*N*_ satisfying condition (6) and *β* satisfying (8), the iterative solution of model (5) leads to

10 $$ {\lim}_{k\rightarrow \infty}I_{k}=I+o(N),  $$

where *I* is the noise free solution(ground truth) and *o*(*N*) denotes the remaining noise with the variance far less than the variance of the noise *N*.

#### *Proof*

Let *F*_*k*_=1/*ζ*_*uv*_(1−(1−*β*|*ζ*_*uv*_|^2^)^*k*^) be the noise amplification factors for *k*=1,2…. Known from lemma (1), *F*_*k*_ is infinite so there must be a minimum number *k* satisfying

11 $$ \text{Var}\left(\sum\limits_{u,v}\left(\frac{N}{F_{k}},Z_{{uv}}\right)Z_{{uv}}\right)<\text{Var}(N)  $$

when noise *N* is bounded. Here, we suppose *k*=1 for convenience though this number depends the eigenvalues of *P*. Thus, we start with the iteration solution(4) for *k*=1,

12 $$\begin{array}{*{20}l} I_{1}& = I_{0}+\beta P^{T}\left(J-{PI}_{0}\right)\\ &=\sum\limits_{u,v}\left(1-\left(1-\beta|\zeta_{{uv}}|^{2}\right)\right)\left(I,Z_{{uv}}\right)Z_{{uv}} \\ &\quad+\sum\limits_{u,v}\frac{1}{\zeta_{{uv}}}\left(1-\left(1-\beta|\zeta_{{uv}}|^{2}\right)\right)\left(N,Z_{{uv}}\right)Z_{{uv}}\\ &=D_{1}+N_{1}, \end{array} $$

where *I*_0_ is the initial image and *D*_1_ is the first sum on right side. The first term in (12) is noise-free iterative solution while the second term is noise contribution with the factor *F*_1_=*β* *ζ*_*uv*_. Since most of the eigenvalues have absolute values small than 1, Var(*N*_1_)∝Var(*N*). Then we apply the filter (6) to (12) and have

13 $$ I_{1}=R_{N}(I_{1})=R_{N}(D_{1}+N_{1})=D_{1}+o(N_{1}).  $$

The noise is now reduced by a factor *Δ*≪1 according to (6), i.e., Var(*o*(*N*_1_))∝*Δ*Var(*N*_1_)∝*Δ* *σ* ^2^, where *σ* ^2^ is the variance of noise *N*.

The intensity of image *I*_1_ can be rewritten

14 $$\begin{array}{*{20}l} I_{1}&=D_{1}+o\left(N_{1}\right)\\ &=\sum\limits_{u,v}\left(1-\left(1-\beta|\zeta_{{uv}}|^{2}\right)\right)\left(I,Z_{{uv}}\right)Z_{{uv}}\\ &+\sum\limits_{u,v}\frac{1}{\zeta_{{uv}}}\left(1-\left(1-\beta|\zeta_{{uv}}|^{2}\right)\right)\left(\frac{o(N_{1})}{F_{1}},Z_{{uv}}\right)Z_{{uv}}, \end{array} $$

where $o(N_{1})={\sum \nolimits }_{{uv}} (o(N_{1}),Z_{{uv}})Z_{{uv}} ={\sum \nolimits }_{{uv}}F_{1} (o(N_{1})/F_{1}, Z_{{uv}})Z_{{uv}}$ is used and *F*_1_ is defined at the beginning of the proof.

From (14) and (4), we give the iteration solution for *k*=2

$$\begin{array}{*{20}l} I_{2} & = I_{1}+\beta P^{T}(J-{PI}_{1})\\ &=I_{1}+\beta P^{T}\left(PI+\sum\limits_{u,v}\left(\frac{o(N_{1})}{F_{1}},Z_{{uv}}\right)Z_{{uv}}-n{1}\right)\\ &\quad+\beta P^{T}\left(N-\sum\limits_{u,v}\left(\frac{o(N_{1})}{F_{1}},Z_{{uv}}\right)Z_{{uv}}\right)\\ &=\sum\limits_{u,v}\left(1-\left(1-\beta|\zeta_{{uv}}|^{2}\right)^{2}\right)\left(I,Z_{{uv}}\right)Z_{{uv}}\\ &\quad+\sum\limits_{u,v}\frac{1}{\zeta_{{uv}}}\left(1-\left(1-\beta|\zeta_{{uv}}|^{2}\right)^{2}\right)\left(\frac{o(N_{1})}{F_{1}},Z_{{uv}}\right)Z_{{uv}}\\ &\quad+\beta P^{T}\left(N-\sum\limits_{u,v}\left(\frac{o(N_{1})}{F_{1}},Z_{{uv}}\right)Z_{{uv}}\right)\\ &=D_{2}+\sum\limits_{u,v}\frac{F_{2}}{F_{1}}\left(o\left(N_{1}\right),Z_{{uv}}\right)Z_{{uv}}\\ &\quad+\beta P^{T}\left(N-\sum\limits_{u,v}\left(\frac{o(N_{1})}{F_{1}},Z_{{uv}}\right)Z_{{uv}}\right)\\ &=D_{2}+N_{2}. \end{array} $$

where *N*_2_ is the sum of the two noise terms on the right side. By (11) and lemma (1), 1≤*F*_2_/*F*_1_<2, Var(*N*_2_)∝Var(*N*)=*σ* ^2^ is obtained.

Repeating in the operation (13) we have

$$ I_{2}=R_{N}(I_{2})=R_{N}(D_{2}+N_{2})=D_{2}+o(N_{2}), $$

where the variance of *o*(*N*_2_) is far less than that of *N*_2_, also far less than that of *N*.

In general, we have

$$I_{k}=D_{k}+N_{k}, $$

where

$$\begin{aligned} D_{k}=\sum\limits_{u,v}\left(1-\left(1-\beta|\zeta_{{uv}}|^{2}\right)^{k}\right)\left(I,Z_{{uv}}\right)Z_{{uv}} \end{aligned} $$

and

$$\begin{aligned} N_{k}&=\sum\limits_{{uv}}\frac{F_{k}}{F_{k-1}}\left(o\left(N_{k-1}\right),Z_{{uv}}\right)Z_{{uv}}\\ &\quad+\beta P^{T}\left(N-\sum_{{uv}}\left(\frac{o(N_{k-1})}{F_{k-1}},Z_{{uv}}\right)Z_{{uv}}\right). \end{aligned} $$

By applying *R*_*N*_ to *I*_*k*_, we get the iterative image

$$I_{k}=R_{N}(I_{k})=D_{k}+o(N_{k}), $$

where Var(*o*(*N*_*k*_))∝*Δ*Var(*N*)=*Δ* *σ* ^2^. Therefore, the iterative solutions converge to the real scene and the noise is controlled to the order of *Δ* *σ* ^2^ in the iterative process. □

It can be seen from the above derivation that the separation of structure recovery from noise amplification in the VanCittert expression is the key that enables us to express noise amplification factor in the *k*th iteration to be *F*_*k*_/*F*_*k*−1_, which is always between 1 and 2 for all *k*. This makes it possible to control the noise amplification over a finite number of iterations by a noise reduction operator satisfying (6). Consequently, the iterative solution converge to the ground truth with higher-order infinitesimal noise.

## Results and discussion

In this section, we undertake two experiments to test our method and compare the results with existing methods. The first experiment contains two images, ’Cameraman256.png’ and ’Lena512.tif’, that are commonly used for measuring efficiency of algorithms for structure restoration because they contain elaborate structures, such as lines, buttons, and textures. These images are the subjects of a recent extensive investigation by an iterative decoupled deblurring BM3D algorithm (IDD-BM3D) [6], which is formulated based on the Nash equilibrium balance of two objective functions undertaking separate denoising and deblurring operations. IDD-BM3D has showed state of the art restoration performance compared to seven other existing methods, which include Fourier-Wavelet regularized deconvolution (ForWaRD) [15], space-variant Gaussian scale mixtures (SV-GCM) [16], shape-adaptive discrete cosine transform(SA-DCT) [17], BM3D deblurring (BM3DDEB) [3], analysis-based sparsity (L0-Abs) [18], adaptive total variation image deblurring by a majorization minimization approach (TVMM) [19], and finally a method based on spatially weighted total variation (CGMK) [20]. We test on the same six scenarios in [6], which have different PSF shapes and blurring strengths as well as noise levels listed in Table 1. Comparisons with all the eight methods are made quantitatively through the measurement of peak signal-to-noise ratio (PSNR).

**Table 1:** PSF and noise variation for each scenario

Scenario	Blur PSF	*σ* ^2^
1	1/(1+*x* ^2^+*y* ^2^),*x*,*y*=−7,…,7	2
2	1/(1+*x* ^2^+*y* ^2^),*x*,*y*=−7,…,7	8
3	9×9 uniform	0.3
4	[1 4 6 4 1]^*T*^[1 4 6 4 1]/256	49
5	Gaussian with std=1.6	4
6	Gaussian with std=0.4	64

As discussed earlier, we choose BM3D (http://www.cs.tut.fi/~foi/GCF-BM3D) as our noise reduction filter because it combines the transform-domain filter [13] with non-local mean filter [14] and has shown improved performance over the both methods individually on their own. Our method is easy to operate, requiring only two parameters: noise standard deviation, *σ*, as an input for BM3D denoising and the step interval, *s*, between two neighboring denoising operations in the iteration (denoising is not necessary for each iteration step for efficient computing). In general, the two parameters depend on the levels of blur and noise in an observation (input image). We have found that our method can produce good performance in a large area in the two parameter space, showing the robustness of the method against the setting of the two parameters. The solutions converge around 1200 iterations for all scenarios except scenario 3 which requires 10,000 iterations because of severe blur in this scenario. Due to high noise levels in scenarios 4 and 6, BM3D is applied to the observations before our iterative algorithm is implemented. To investigate the effects of different denoising algorithms on the performance of our method, we have also implemented the wavelet domain shrinking filter *T*_SW_ as an alternative denoising operator in our method. Table 2 shows the results of PSNR for our algorithm, both with BM3D and *T*_SW_, and the eight existing methods, the latter are from [6]. From the table, we can conclude that our method with BM3D outperforms the existing seven methods for both images under different scenarios and is not far behind the state-of-the-art IDD-BM3D. As expected, the algorithm with *T*_SW_ performs not as good as that with BM3D, because the latter is better than the former as a denoising method.

**Table 2:** PSNR of the methods in six scenarios

	Scenarios						Scenarios					
	1	2	3	4	5	6	1	2	3	4	5	6
Methods	Cameraman (256×256)						Lena (512×512)					
Input PSNR	22.23	22.16	20.76	24.62	23.36	29.82	25.61	25.46	24.11	28.06	27.81	29.98
ForWaRD [15]	28.99	27.24	28.10	27.02	26.50	33.74	33.30	31.94	32.81	31.74	32.66	35.45
SV-GSM [16]	29.68	27.71	28.09	27.35	26.61	34.01	-	-	-	-	-	-
SA-DCT [17]	30.34	28.49	29.31	27.99	27.08	34.53	34.8	33.14	33.63	33.3	33.24	35.87
BM3DDEB [3]	30.42	28.56	29.1	27.96	27.09	34.52	35.2	33.57	33.81	33.62	33.53	36.43
L0-Abs [18]	29.93	27.93	29.72	27.61	26.94	33.21	33.91	32.75	33.63	32.9	33.38	31.96
TVMM [19]	29.64	27.33	29.3	27.19	26.72	31.12	33.61	32.02	33.31	32.33	32.77	32.82
CGMK [20]	30.03	27.65	29.91	27.42	26.9	33.15	34.01	32.41	33.7	32.3	33.09	34.49
IDD-BM3D [6]	*31.08*	*29.28*	*31.21*	*28.60*	*27.67*	*34.71*	35.22	*33.65*	*34.75*	*33.78*	*34.01*	36.37
Ours(BM3D)	30.73	28.71	30.57	28.25	27.43	34.65	*35.29*	33.41	34.53	33.70	33.91	*36.46*
Ours(*T*_SW_)	28.62	26.52	27.99	26.18	26.04	32.23	33.90	32.38	32.85	32.43	32.87	34.27

The italicized values in this table indicate the method which leads to the best result among all compared methods

**Fig. 1:**
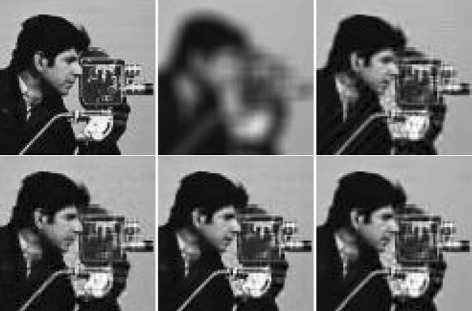
Deblurring of Cameraman image, scenario 3. From *left* to *right* and from *top* to *bottom* are presented *zoomed* fragments of the following images: *original*, *blurred*, and *noisy*, reconstructed by BM3DDEB, L0-Abs, IDD-BM3D, and our method. In our method, the two input parameters used, (*σ*,*s*), are (7.5,550) for this scenario, (7.5,85) for scenario 1, (7.5,25) for scenario 2, (7.5,10) for scenario 4, (7.5,50) for scenario 5, and (7.5,5) for scenario 6

**Fig. 2:**
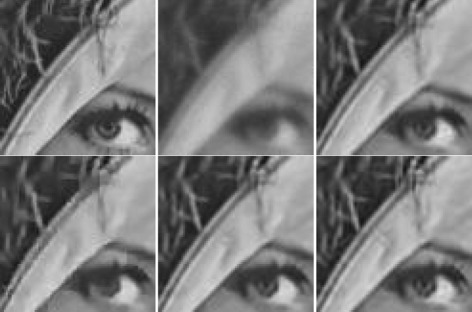
Deblurring of Lena image, scenario 2. From *left* to *right* and from *top* to *bottom* are presented *zoomed* fragments of the following images: *original*, *blurred*, and *noisy*, reconstructed by BM3DDEB, L0-Abs, IDD-BM3D, and our method. In our method, the two input parameters used, (*σ*,*s*), are (7.5,25) for this scenario, (7.5,85) for scenario 1, (7.5,550) for scenario 3, (7.5,10) for scenario 4, (7.5,50) for scenario 5, and (7.5,5) for scenario 6

We have further investigated the above results by using the structural similarity (SSIM) index matric, which is a method for measuring the structural similarity between two images. The results are shown in Table 3. As seen from the table, L0-Abs, BM3DDEB, and our methods both with BM3D and *T*_SW_ have all performed better than IDD-BM3D in terms of SSIM for the first five scenarios, although IDD-BM3D gives the highest PSNR values as discussed above. By comparing the results in Tables 2 and 3, it is pleasing to see that our method with BM3D gives very good and balanced performance in terms of both noise reduction and structure preservation. Figures 1 and 2 show the restored images by four methods used. As seen by visual inspection, BM3DDEB produces some obvious artifacts around the edge of cameraman and L0-Abs cannot restore some details of the eye in the image of Lena because of noise. In comparison, IDD-BM3D and our method with BM3D denoise very well and our method shows better recovery of elaborative features in these images.

**Table 3:** SSIM of the four methods in six scenarios

	Scenarios					
	1	2	3	4	5	6
Methods	Cameraman (256×256)					
Input SSIM	0.93	0.93	0.92	0.96	0.95	0.98
L0-Abs	0.99	0.98	0.99	0.98	0.98	0.99
BM3DDEB	0.99	0.99	0.99	*0.98*	0.98	*0.99*
IDD-BM3D	0.96	0.94	0.95	0.96	0.96	0.99
Ours(BM3D)	*0.99*	*0.99*	*0.99*	0.98	*0.98*	0.99
Ours(*T*_SW_)	0.97	0.97	0.97	0.96	0.96	0.99

The italicized values in this table indicate the method which leads to the best result among all compared methods

**Fig. 3:**
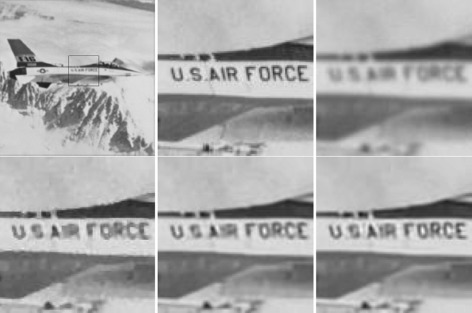
Deblurring of Jetplane image, scenario 5. From *left* to *right* and from *top* to *bottom* are the following images: *blurred* and *noisy*, fragments of *original*, *blurred,* and *noisy*, results of Fnnmm, IDD-BM3D, and ours. In our method (fixed parameters), the two input parameters, (*σ*,*s*), are (7.5,50) for this scenario, (7.5,85) for scenario 1, (7.5,25) for scenario 2, (7.5,550) for scenario 3, (7.5,10) for scenario 4, and (7.5,5) for scenario 6. In our method with automated parameter setting, *σ*_thr_=3

We have further developed an automated parameter setting procedure for our method. Here, we first estimate the noise standard deviation in the observation and set this value as the denoising threshold, *σ*_*thr*_. During the iteration, the noise level of the image is estimated at each iteration step and when it exceeds *σ*_*thr*_, then BM3D denoising is applied. The procedure is simple to operate, which can be important for real scene applications. We used this procedure in the second experiment of Jetplane.png, and the result are compared with those of IDD-BM3D and the fast non-convex non-smooth method (Fnnmm) method [21] on PSNR and text restorations. Fnnmm introduces non-convex functions applied on discrete total variation as a regularization and provides fast algorithms to minimize the energy function. The code of Fnnmm is downloaded at http://www.math.hkbu.hk/~mng/imaging-software.html. We used the same six scenarios as in the first experiment, which in turn allows us to use the same parameter setting for IDD-BM3D. For Fnnmm, we fix parameter *α*_*ep*_ to be 0.5 as given in the paper and scan the other parameter for each of the six scenarios for the highest possible value of PSNR. The PSNR values of restored images are given in Table 4. As seen in the table, our method with automated parameter setting, IDD-BM3D and our method with fixed parameter setting achieved 3, 2, and 1 best values out of the 6 scenarios, respectively, but differences among them are small and all of them are significantly better than those of Fnnmm. Figure 3 shows a zoomed area of the body of the plane. Our results show slightly better resolution on some of the letters on the plane.

**Table 4:** PSNR of the three methods in six scenarios

	Scenarios					
	1	2	3	4	5	6
Methods	Jetplane (512×512)					
Input PSNR	24.98	24.84	23.43	27.31	26.84	29.85
Fnnmm [21]	33.10	30.89	32.97	30.76	30.60	34.91
IDD-BM3D [6]	35.43	*33.41*	*34.58*	32.63	32.12	36.60
Ours (fixed parameters)	35.03	32.84	34.57	32.91	32.04	*36.65*
Ours (automated parameters)	*35.44*	33.34	34.29	*33.35*	*32.42*	36.27

The italicized values in this table indicate the method which leads to the best result among all compared methods

**Fig. 4:**
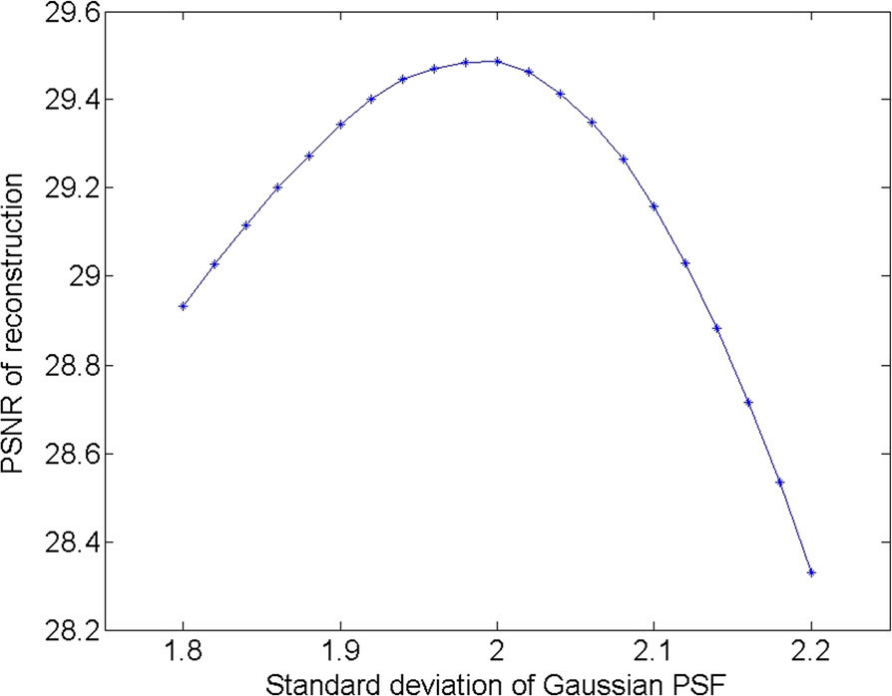
PSNR of reconstruction with different PSFs

Finally, we test the robustness of our method against the fluctuations of the size of PSF in the model, since the exact value is usually unknown in practice and is estimated. For this, we undertake the experiment on image Jetplane.png, which is blurred by a Gaussian PSF of standard deviation *σ*=2 and noise level of 40 db. We measure PSNR of the restored images on varying *σ* by ±10*%* from the exact value. As shown in Fig. 4, PSNR decreases from its peak value of 29.5 on both sides, dropping faster when *σ* is larger, due to more noticeable artifacts of the hard shoulder and plunge effects around an edge in this situation. As a result, the restored image looks to artificially have a higher contrast when *σ* is larger than the exact value. Overall, the reduction of PSNR is 2 and 4% by varying the standard deviation 10% smaller or larger than the exact value, respectively.

## Conclusions

In summary, we have developed a new robust iterative method for image restoration in which an iterative cost function is utilized to optimize the gradient in the steepest descent by adaptively adjusting to the current state in the iterative process. We show that the iterative solution converges to the real scene despite noise contamination in an observation, and the restoration error can be controlled by an order of magnitude smaller than the noise level in the observation. Different from the well-established regularization methods, which introduce a penalty to solve the ill-posed problem, we directly apply VanCittert algorithm to minimize residual along gradient for structure restoration and to suppress noise through a noise reduction operator. It turns out to be a denoising problem in an iterative manner, and the noise can be removed judiciously by applying existing noise reduction methods. We have undertaken two numerical experiments to investigate the performance of this method and compare to existing regularization methods. We show that our method performs to the level close to the best of the methods currently available in terms of recovering elaborate structures and reducing noise, and favorably compared to the many other existing methods. Moreover, our method requires simple parameter setting, particularly in the second experiment where a single parameter is estimated from the observation. This could be a great advantage for real-world applications. We note finally that we have only considered additive noise in this paper, for images contaminated by multiplicative noise, some newly developed noise reduction filters, such as [22] may be applied, which can be our future work in this area.

## Abbreviations

BM3D: Block matching 3-D
CGMK: Chantas, Galatsanos, Molina, Katasaggelos
Fnnmm: Fast non-convex non-smooth method
ForWaRD: Fourier-wavelet regularized deconvolution
IDD-BM3D: Iterative decoupled deblurring block matching 3-D
L0-Abs: *L*_0_ Analysis-based sparsity
NLM: Non-local mean
PSF: Point spread function
PSNR: Peak signal-to-noise ratio
SA-DCT: Shape-adaptive discrete cosine transform
SV-GCM: Space-variant Gaussian scale mixtures
TVMK: Total variation image deblurring by a majorization minimization
